# Optimization of heterologous DNA-prime, protein boost regimens and site of vaccination to enhance therapeutic immunity against human papillomavirus-associated disease

**DOI:** 10.1186/s13578-016-0080-z

**Published:** 2016-02-25

**Authors:** Shiwen Peng, Jin Qiu, Andrew Yang, Benjamin Yang, Jessica Jeang, Joshua W. Wang, Yung-Nien Chang, Cory Brayton, Richard B. S. Roden, Chien-Fu Hung, T.-C. Wu

**Affiliations:** Department of Pathology, Johns Hopkins Medical Institutions, Baltimore, MD USA; Department of Obstetrics and Gynecology, Shanghai Tenth People’s Hospital of Tongji University, Shanghai, China; Department of Molecular and Comparative Pathobiology, Johns Hopkins Medical Institutions, Baltimore, MD USA; Department of Pathology, Department of Gynecology and Obstetrics, and Department of Oncology, Johns Hopkins Medical Institutions, Baltimore, MD USA; Department of Pathology and Department of Oncology, Johns Hopkins Medical Institutions, Baltimore, MD USA; Departments of Pathology, Department of Obstetrics and Gynecology, Department of Molecular Microbiology and Immunology, and Department of Oncology, Johns Hopkins Medical Institutions, CRBII Room 309, 1550 Orleans Street, Baltimore, MD 21231 USA

**Keywords:** Human papilloma virus, HPV, TA-CIN, pNGVL4a-Sig/E7(detox)/HSP70, pNGVL4a-CRT/E7(detox), pNGVL4a-CRT-E6E7L2, Prime-boost

## Abstract

**Background:**

Human papillomavirus (HPV) has been identified as the primary etiologic factor of cervical cancer as well as subsets of anogenital and oropharyngeal cancers. The two HPV viral oncoproteins, E6 and E7, are uniquely and consistently expressed in all HPV infected cells and are therefore promising targets for therapeutic vaccination. Both recombinant naked DNA and protein-based HPV vaccines have been demonstrated to elicit HPV-specific CD8+ T cell responses that provide therapeutic effects against HPV-associated tumor models. Here we examine the immunogenicity in a preclinical model of priming with HPV DNA vaccine followed by boosting with filterable aggregates of HPV 16 L2E6E7 fusion protein (TA-CIN).

**Results:**

We observed that priming twice with an HPV DNA vaccine followed by a single TA-CIN booster immunization generated the strongest antigen-specific CD8+ T cell response compared to other prime-boost combinations tested in C57BL/6 mice, whether naïve or bearing the HPV16 E6/E7 transformed syngeneic tumor model, TC-1. We showed that the magnitude of antigen-specific CD8+ T cell response generated by the DNA vaccine prime, TA-CIN protein vaccine boost combinatorial strategy is dependent on the dose of TA-CIN protein vaccine. In addition, we found that a single booster immunization comprising intradermal or intramuscular administration of TA-CIN after priming twice with an HPV DNA vaccine generated a comparable boost to E7-specific CD8+ T cell responses. We also demonstrated that the immune responses elicited by the DNA vaccine prime, TA-CIN protein vaccine boost strategy translate into potent prophylactic and therapeutic antitumor effects. Finally, as seen for repeat TA-CIN protein vaccination, we showed that the heterologous DNA prime and protein boost vaccination strategy is well tolerated by mice.

**Conclusions:**

Our results provide rationale for future clinical testing of HPV DNA vaccine prime, TA-CIN protein vaccine boost immunization regimen for the control of HPV-associated diseases.

**Electronic supplementary material:**

The online version of this article (doi:10.1186/s13578-016-0080-z) contains supplementary material, which is available to authorized users.

## Background

Infection with a high-risk human papillomavirus (hrHPV) type, especially HPV16, is the primary cause of 5 % of all cancers worldwide. Cervical cancer is the fourth most deadly women’s cancer worldwide [1] and 99 % of cases are associated with hrHPV infection. Overall, 50–60 % of cervical cancer is associated with HPV16 and ~20 % with HPV18 [2]. A subset of other anogenital and ororpharyngeal cancers are also associated with hrHPV, primarily HPV16. Currently, there is no HPV-targeted antiviral treatment for persistent genital infection and low-grade squamous intraepithelial lesions (LSIL). Although most HPV16 infections spontaneously resolve, repeat screening for high-grade squamous intraepithelial lesions (HSIL) is recommended. Moreover, while surgical treatment is quite effective for precancer and localized early cancer lesions of the cervix, surgical treatment of vaginal, vulval and anal high-grade intraepithelial lesions is associated with significant morbidity and high recurrence rates [3, 4]. Thus, there is a clear need for treatments to clear HPV16 and other high-risk HPV infections and associated diseases. Immunotherapy targeting the E6 and/or E7 viral proteins has particular promise because HPV E6 and E7 are functionally required for the initiation and maintenance of the disease, and both represent non-‘self’, foreign antigens which are not subject to central immune tolerance [5].

Vaccines based on naked DNA have promise as an approach for the control of HPV due to their promising safety record and ability to present viral antigens through the major histocompatibility complex (MHC) class I pathway as well as their practicality due to relative simplicity of manufacture and stability. We have previously developed a candidate therapeutic HPV vaccine, pNGVL4a-Sig/E7(detox)/HSP70, comprising a naked DNA vector that expresses a tandem fusion of signal peptide (sig), HPV16 E7 antigen and *Mycobacterium tuberculosis* heat shock protein 70 (HSP70), which by virtue of its fusion elicits potent E7-specific, and CD8 T cell driven antitumor immunity [6]. Intramuscular (i.m.) administration of pNGVL4a-Sig/E7(detox)/HSP70 DNA was well tolerated by patients with HPV16 + CIN2/3 [7, 8]. However, in comparison to the murine models, vaccination with this construct in humans elicited weaker systemic E7-specific CD8+ T cell responses that did not directly correlate with lesion regression [7, 9]. A potential reason may be the less efficient in vivo transduction (and consequently low antigen expression) in humans compared to mice after i.m. injection of a naked DNA vaccine. Heterologous prime-boost vaccination is a means of priming the immune system by administration of a target antigen via one type of vector, with subsequent boosting of immunologic memory by re-administration of the antigen in the context of a different vector that optimally confers higher antigen levels than during priming. A previous trial utilized pNGVL4a-Sig/E7(detox)/HSP70 DNA as a priming vaccine and followed by a boost with the recombinant vaccinia virus TA-HPV that expresses E6 and E7 of both HPV16 and HPV18 [8].

DNA-based priming vaccination followed by recombinant protein booster immunization with relevant soluble antigens has been shown to be well tolerated and elicited both cellular and humoral immune responses in HIV and malaria infected patients [10–13]. TA-CIN is a single fusion protein comprised of HPV16 E6, E7 and L2 proteins linked in tandem that forms a filterable aggregated antigen and has potential as a candidate preventive and therapeutic HPV vaccine. Vaccination with L2 can confer humoral immunity against a broader range of papillomavirus types in animal models, as compared to the type-restricted immunity observed with L1 virus-like particle (VLP) vaccines [14]. Importantly, vaccination of HPV16 infected-patients with TA-CIN is also designed to trigger therapeutic immunity targeting the E6 and E7 of HPV16. A phase I trial provided preliminary evidence that serial intramuscular vaccination with TA-CIN in the absence of an adjuvant is safe, well-tolerated, and immunogenic in healthy volunteers [15]. Other trials have explored TA-CIN protein as a priming or a booster vaccine and have shown that intramuscular immunization with TA-CIN after either TA-HPV or topical imiquimod administration is safe and generates E7-specific CD8+ T cell responses [16, 17]. However the use of TA-CIN recombinant protein as a booster vaccine following priming with a naked DNA vaccine has not been tested.

In the current study, we investigated in mice the immunogenicity of priming with the pNGVL4a-Sig/E7(detox)/HSP70 DNA vaccine followed by boosting with TA-CIN while exploring the optimal approach for their combination, and the impact of intra-muscular versus intra-dermal delivery of TA-CIN.

## Results

### Optimization of the DNA prime and protein boost vaccine regimen for the induction of E7-specific CD8+ T cell immunity

**Fig. 1:**
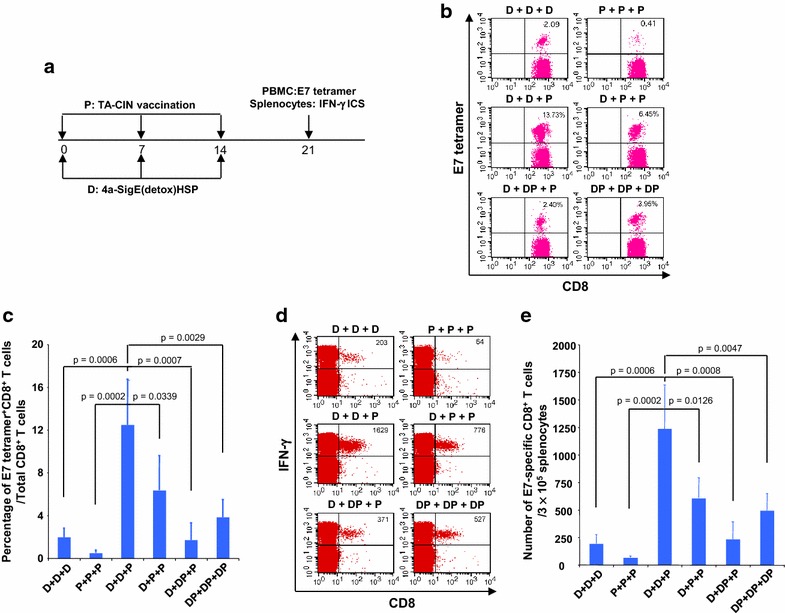
Comparison of HPV16 E7-specific CD8+ T cell responses induced by different combination of pNGVL4a-Sig/E7(detox)/HSP70 DNA vaccine and TA-CIN vaccination. Five to eight weeks old female C57BL/6 mice (5 mice/group) were vaccinated with 1) 25 μg/mouse of pNGVL4a-Sig/E7(detox)/HSP70 DNA in 50 μl via intramuscular injection (leg muscle) three times with 1-week intervals between each vaccination (D + D + D); 2) 25 μg/mouse of TA-CIN in 20 μl via i.d. injection (lower back) three times with 1-week intervals between each vaccination (P + P + P); 3) 25 μg/mouse of pNGVL4a-Sig/E7(detox)/HSP70 DNA in 50 μl via intramuscular injection twice followed by once with 25 μg/mouse of TA-CIN in 20 μl via i.d. injection with 1-week intervals between each vaccination (D + D + P); 4) 25 μg/mouse of pNGVL4a-Sig/E7(detox)/HSP70 DNA in 50 μl via intramuscular injection once followed by twice with 25 μg/mouse of TA-CIN in 20 μl via i.d. injection with 1-week intervals between each vaccination (D + P + P); 5) 25 μg/mouse of pNGVL4a-Sig/E7(detox)/HSP70 DNA in 50 μl via intramuscular injection once, followed by 25 μg/mouse of pNGVL4a-Sig/E7(detox)/HSP70 DNA in 50 μl via intramuscular injection +25 μg/mouse of TA-CIN in 20 μl via i.d. injection concomitantly once, followed by 25 μg/mouse of TA-CIN in 20 μl via i.d. injection one time, with 1-week intervals between each set of vaccinations (D + DP + P); and 6) 25 μg/mouse of pNGVL4a-Sig/E7(detox)/HSP70 DNA in 50 μl via intramuscular injection +25 μg/mouse of TA-CIN in 20 μl via i.d. injection concomitantly three times with 1-week interval between each set of vaccinations (DP + DP + DP). Seven days after last vaccination, PBMCs were prepared and stained with anti-mouse CD8 and HPV16 E7 tetramer. Splenocytes were prepared and stimulated with 1 μg/ml of HPV16 E7aa49–57 peptide at the presence of GolgiPlug (1 μl/ml) overnight at 37 °C and stained with anti-mouse CD8 followed by intracellular IFN-γ. The data were acquired with FACSCalibur and analyzed with CellQuest. **a** Schematic illustration of the experiment. **b** and **c** Flow cytometry analysis of HPV16 E7-specific CD8+ T cells in peripheral blood. **d** and **e** Flow cytometry analysis of HPV16 E7-specific CD8+ T cells in spleen

Both the TA-CIN protein and the pNGVL4a-Sig/E7(detox)/HSP70 DNA have been administered intra-muscularly to patients with minimal side effects. However, the systemic HPV-specific CD8+ T cell responses were difficult to detect in each case. We hypothesized that a prime-boost regimen of pNGVL4a-Sig/E7(detox)/HSP70 DNA followed by TA-CIN protein could generate more potent systemic E7-specific CD8+ T cell responses. To determine the appropriate regimen, C57BL/6 mice (5 per group) were vaccinated with intra-muscular 25 µg pNGVL4a-Sig/E7(detox)/HSP70 DNA injection and/or intra-dermal 25 µg TA-CIN protein injection each time for a total of three times, with each immunization spaced by a 1 week interval (Fig. 1a). One week after last vaccination, PBMC and splenocytes of mice were collected, and the E7-specific CD8 T cells responses generated by various regimens were compared. As shown in Fig. 1b and c, mice vaccinated with pNGVL4a-Sig/E7(detox)/HSP70 DNA twice followed by a single TA-CIN protein boost generated the highest percentage of E7-specific tetramer labeled CD8+ T cells in peripheral blood compared to mice vaccinated with other regimens including administration of both vaccines concomitantly (DP) up to three times. Furthermore, this regimen also elicited the highest number of splenic IFNγ + E7-specific CD8+ T cells (Fig. 1d, e). These results suggest that heterologous vaccination by priming with two repeat doses of pNGVL4a-Sig/E7(detox)/HSP70 DNA followed by a single TA-CIN protein vaccine boost is the most immunogenic regimen in mice.

### Intra-muscular and intra-dermal TA-CIN protein booster immunization following priming with E7 DNA vaccination generate comparable potent E7-specific CD8+ T cell mediated immune responses

**Fig. 2:**
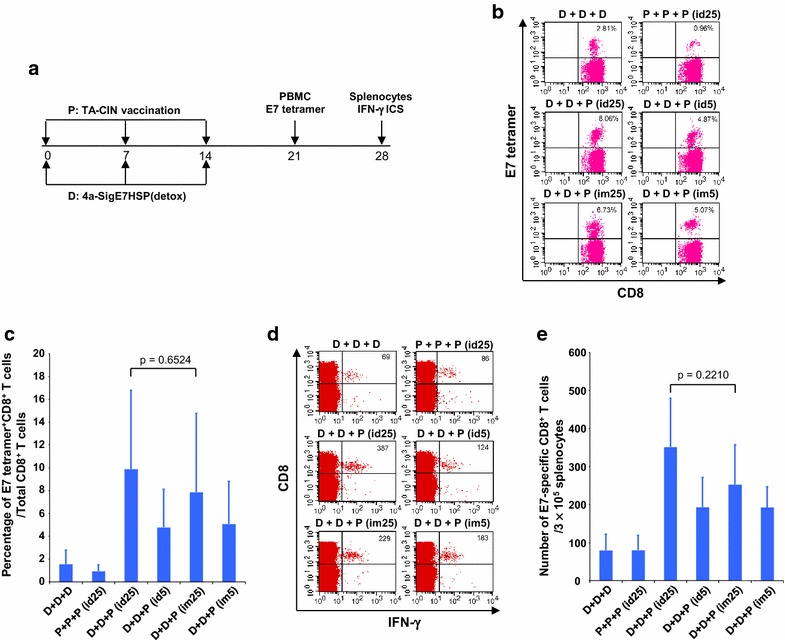
Comparison of HPV16 E7-specific CD8+ T cell responses induced by TA-CIN vaccination at different dose and vaccination route when combined with pNGVL4a-Sig/E7(detox)/HSP70 DNA vaccination. Five to eight weeks old female C57BL/6 mice (5 mice/group) were vaccinated with either 25 μg/mouse of pNGVL4a-Sig/E7(detox)/HSP70 DNA in 50 μl via intramuscular injection (leg muscle) or indicated dose of TA-CIN in 20 μl via either i.d. or i.m. injection. The mice were primed as indicated twice with 1-week interval. Seven days after last vaccination, PBMCs were prepared and stained with anti-mouse CD8 and HPV16 E7 tetramer. Splenocytes were prepared and stimulated with 1 μg/ml of HPV16 E7aa49–57 peptide at the presence of GolgiPlug (1 μl/ml) overnight at 37 °C. The cells were then stained with anti-mouse CD8 followed by intracellular IFN-γ. The data were acquired with FACSCalibur and analyzed with CellQuest. **a** Schematic illustration of the experiment. **b** and **c** Flow cytometry analysis of HPV16 E7-specific CD8+ T cells in peripheral blood. **d** and **e** Flow cytometry analysis of HPV16 E7-specific CD8+ T cells in spleen

We sought to determine whether the dose and route of TA-CIN booster vaccination affects the generation of antigen-specific CD8+ T cell responses after priming with two intra-muscular vaccinations with 25 µg pNGVL4a-Sig/E7(detox)/HSP70 DNA. C57BL/6 mice (5 per group) were first vaccinated twice via intra-muscular administration of 25 µg pNGVL4a-Sig/E7(detox)/HSP70 DNA, and then boosted with either 5 or 25 µg TA-CIN vaccination administered either intra-dermally (id) or intramuscularly (im), using the schedule described in Fig. 2a. As shown in Fig. 2b and c, mice, whether boosted with TA-CIN intra-dermally or intra-muscularly, generated a comparable percentage of E7-specific CD8+ T cells in peripheral blood 1 week after completion of vaccination. When a lower dose of TA-CIN (5 µg) was used, the responses were again similar, although lower than the response generated by the 25 µg booster dose (Fig. 2b, c). As shown in Fig. 2d and e, when the number of splenic IFNγ + E7-specific CD8+ T cells was compared for the same mice at 2 weeks after vaccination, a similar pattern was observed.

We further assessed the dosage effect in boosting of E7-specific CD8+ T cell response upon intramuscular TA-CIN vaccination and demonstrated a dose dependent relationship between the dosages of TA-CIN vaccination to the magnitude of immune responses elicited by vaccination (Additional file 1: Figure S1). However, when we tried to examine the E7-specific CD4+ T cell response generated by the vaccination strategy, no significant E7-specific CD4+ T cell response were observed in treated mice (Additional file 2: Figure S2).

Since TA-CIN administration has been shown to generate HPV 16-specific humoral responses [18], we also verified whether boosting with TA-CIN following priming with pNGVL4a-Sig/E7(detox)/HSP70 DNA can lead to an enhanced HPV-16 E7-specific antibody responses similar to the enhanced E7-specific CD8+ T cell responses. Mice were vaccinated with different pNGVL4a-Sig/E7(detox)/HSP70 DNA and/or TA-CIN regimens using the treatment schedule shown in Additional file 3: Figure S3A. As shown in Additional file 3: Figure S3B, boosting with TA-CIN protein following priming with pNGVL4a-Sig/E7(detox)/HSP70 DNA vaccination generated a similar level of E7-specific antibody response regardless of vaccination route and dosage that is higher than the antibody response generated by homologous pNGVL4a-Sig/E7(detox)/HSP70 DNA vaccination. However, the E7-specific antibody responses generated by the heterologous DNA-prime, TA-CIN protein-boost regimens are slightly lower than the antibody response generated by homologous TA-CIN protein vaccination, suggesting that priming with DNA vaccine followed by boosting with TA-CIN protein vaccine does not enhance the E7-specific humoral responses as effectively as E7-specific CD8+ T cell responses.

Together, these results indicate that TA-CIN booster vaccination by the intra-muscular and intra-dermal routes are similarly effective in generating dose-dependent E7-specific CD8+ T cell immune responses.

### Intra-dermal or intra-muscular TA-CIN boost following DNA vaccine priming lead to the generation of comparable E7-specific CD8+ T cell responses in TC-1 tumor bearing mice

**Fig. 3:**
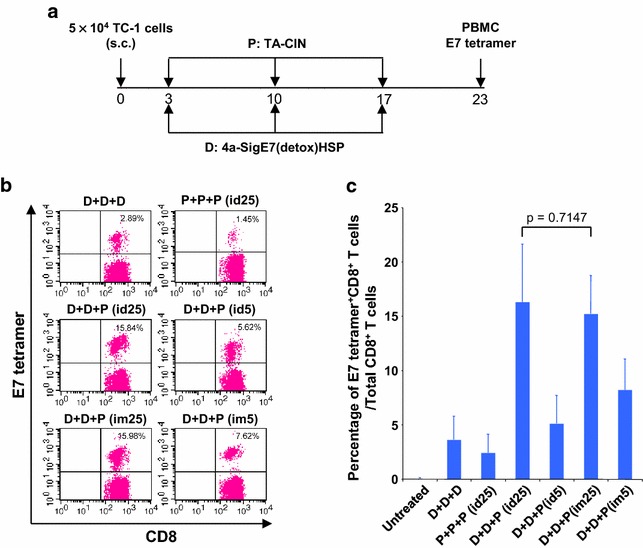
pNGVL4a-Sig/E7(detox)/HSP70 DNA priming followed by TA-CIN boost through either i.d or i.m. injection generated robust HPV16 E7-specific CD8+ T cell response in TC-1 tumor-bearing mice. Five to eight weeks old female C57BL/6 mice (5 mice/group) were injected with 5 × 10^4^ of TC-1 cells subcutaneously on day 0. The mice were vaccinated with either 25 μg/mouse of pNGVL4a-Sig/E7(detox)/HSP70 DNA in 50 μl via intramuscular injection (leg muscle) or TA-CIN with indicated dose in 20 μl via i.d. or i.m. injection. The mice were boosted as indicated twice with 1-week interval. Six days after last vaccination, PBMCs were prepared and stained with anti-mouse CD8 and HPV16 E7 tetramer. The data were acquired with FACSCalibur and analyzed with CellQuest. **a** Schematic illustration of the experiment. **b** and **c** Flow cytometry analysis of HPV16 E7-specific CD8+ T cells in peripheral blood induced by pNGVL4a-Sig/E7(detox)/HSP70 DNA and TA-CIN vaccination

Next, we tested the ability of the DNA prime/TA-CIN boost immunization regimen in eliciting E7-specific CD8+ T cell responses in tumor-bearing mice. C57BL/6 mice (5 per group) were first challenged with TC-1 tumor cells (5x10^4^) subcutaneously. Three days after tumor challenge, mice were vaccinated twice intra-muscularly with 25 µg pNGVL4a-Sig/E7(detox)/HSP70 DNA and boosted once with either 5 or 25 µg TA-CIN protein via the intra-muscular or intra-dermal routes using a treatment schedule described in Fig. 3a. As shown in Fig. 3b and c, tumor-bearing mice that received two pNGVL4a-Sig/E7(detox)/HSP70 DNA (25 µg) priming vaccinations followed by a single boost with the higher dose of TA-CIN protein (25 µg) induced the highest percentage E7-specific CD8+ T cells regardless of administration route, which is consistent with the results observed in naïve mice (Fig. 2b–e).

### TA-CIN protein boost leads to enhanced HPV antigen-specific CD8+ T cell responses following priming with different types of therapeutic DNA vaccines

**Fig. 4:**
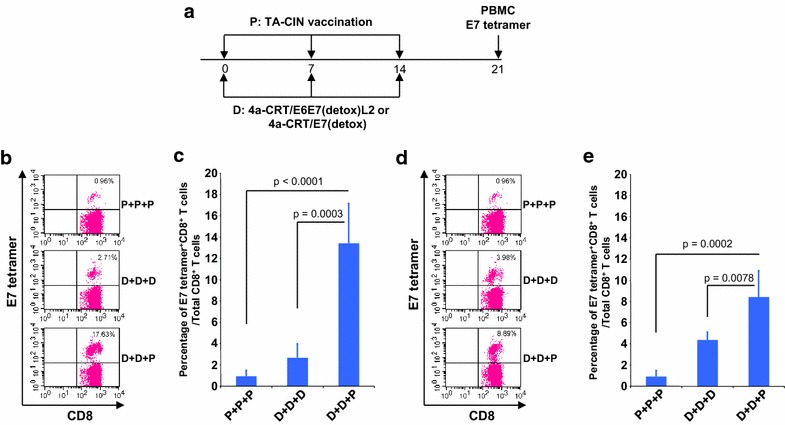
pNGVL4a-CRT-E6E7L2 or pNGVL4a-CRT/E7(detox) DNA prime followed by TA-CIN boost also generated significantly enhanced HPV16 E7-specific CD8+ T cell responses. Five to eight weeks old female C57BL/6 mice (5 mice/group) were vaccinated with either 5 μg/mouse of pNGVL4a-CRT/E7(detox) DNA or pNGVL4a-CRT-E6E7L2 in 50 μl via intramuscular injection (leg muscle) or 25 μg/mouse of TA-CIN in 20 μl via i.d. injection. The mice were boosted as indicated twice with 1-week interval. Seven days after last vaccination, PBMCs were prepared and stained with anti-mouse CD8 and HPV16 E7 tetramer. PBMCs were prepared and stained with anti-mouse CD8 and HPV16 E7 tetramer. The data were acquired with FACSCalibur and analyzed with CellQuest. **a** Schematic illustration of the experiment. **b** and **c** Flow cytometry analysis of HPV16 E7-specific CD8+ T cells in peripheral blood induced by pNGVL4a-CRT/E7(detox) DNA and TA-CIN vaccination. **d** and **e** Flow cytometry analysis of HPV16 E7-specific CD8+ T cells in peripheral blood induced by pNGVL4a-CRT-E6E7L2 DNA and TA-CIN vaccination

There is a clinical trial using a related candidate therapeutic HPV vaccine, pNGVL4a-CRT/E7(detox), that expresses HPV16 E7 as a fusion with human calreticulin (CRT) [9], and a second trial planned using a third candidate therapeutic HPV vaccine, pNGVL4a-CRT-E6E7L2, that fuses E6, E7, and L2 residues 11–200 with CRT. The fusion with CRT greatly enhances MHC class I presentation and the induction of antigen-specific CD8 T cell responses in mice. To assess whether E7-specific CD8+ T cell responses primed by these related therapeutic HPV DNA vaccines can also be further boosted with TA-CIN, a vaccination regimen similar to that used for pNGVL4a-Sig/E7(detox)/HSP70 DNA was used to prime mice with these new DNA vaccines (Fig. 4a). As shown in Fig. 4b and c, mice vaccinated twice with pNGVL4a-CRT/E7(detox) DNA followed by a single TA-CIN booster vaccination generated significantly higher percentage of E7-specific CD8+ T cells compared to mice receiving three DNA or three TA-CIN protein vaccinations. The enhanced E7-specific CD8+ T cell response following a single TA-CIN booster immunization was also observed following priming with pNGVL4a-CRT-E6E7L2 DNA (Fig. 4d, e).

Since both pNGVL4a-CRT-E6E7L2 DNA vaccine and TA-CIN protein vaccine have incorporated multiple HPV antigens in their construct, we also evaluated the generation of immune responses that are specific to these antigens following the administration of these vaccines. As shown in Additional file 4: Figure S4, TA-CIN protein vaccine boost following priming with pNGVL4a-CRT-E6E7L2 DNA vaccine does not generate a higher number of TA-CIN-specific CD4+ T cells or E6-specific CD8+ T cells in mice compared to three doses of pNGVL4a-CRT-E6E7L2 DNA vaccinations or three dose of TA-CIN protein vaccinations.

Furthermore, since pNGVL4a-CRT-E6E7L2 DNA vaccine also encoded L2 protein sequence in its construct, which has been shown to induce HPV16 L2-specific neutralizing antibodies [19], we sought to determine the generation of HPV16 L2-specific antibody response following pNGVL4a-CRT-E6E7L2 DNA and/or TA-CIN protein vaccinations. As shown in Additional file 3: Figure S3D, priming with two pNGVL4a-CRT-E6E7L2 DNA vaccination followed by boosting with one TA-CIN protein vaccination generated a stronger L2-specific antibody response than three pNGVL4a-CRT-E6E7L2 DNA vaccinations, at a level similar to homologous TA-CIN protein vaccination.

Together, these data show that TA-CIN protein can effectively boost the E7-specific CD8+ T cell immune responses of multiple types of HPV E7 DNA-based therapeutic HPV vaccines in C57BL/6 mice.

### TA-CIN protein boost leads to enhanced anti-tumor immunity following priming with therapeutic DNA vaccine

Since a single boost with TA-CIN protein following two priming vaccinations with pNGVL4a-Sig/E7(detox)/HSP70 induced the most robust HPV16 E7-specific CD8+ T cell responses in both naïve and tumor-bearing mice, we therefore examined whether this was also associated with improved anti-tumor immunity against the HPV16 E6E7-expressing TC-1 transplantable tumor model.

**Fig. 5:**
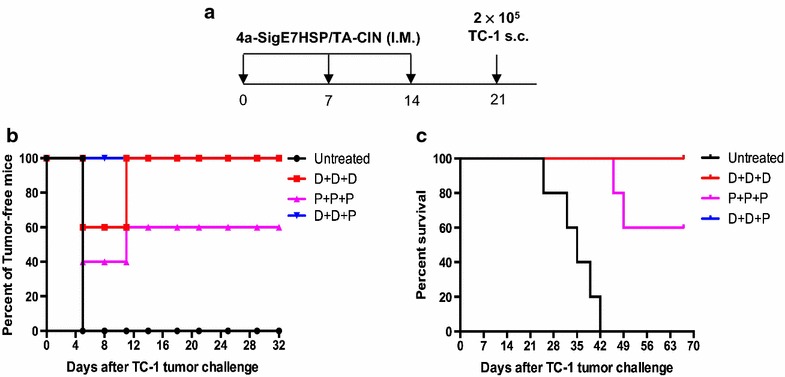
pNGVL4a-Sig/E7(detox)/HSP70 DNA priming followed by TA-CIN boost generated potent protective anti-tumor effects against subsequent TC-1 tumor challenge. One group of 5 ~ 8 weeks old female C57BL/6 mice (5 mice/group) was vaccinated with 25 μg/mouse of pNGVL4a-Sig/E7(detox)/HSP70 DNA in 50 μl three times. Another group of mice was vaccinated with 25 μg/mouse of TA-CIN protein in 20 μl three times. The third group of mice was vaccinated with 25 μg/dose of pNGVL4a-Sig/E7(detox)/HSP70 DNA in 50 μl twice followed by single 25 μg/mouse of TA-CIN protein in 20 μl. All vaccinations were given via intramuscular injection (leg muscle) with 1-week interval. Seven days after the last vaccination, the mice were injected with 2 × 10^5^ of TC-1 tumor cells subcutaneously. **a** Schematic illustration of the experiment. **b** Summary of tumor incidence. **c** Kaplan–Meier survival analysis of TC-1 tumor-bearing mice

We first tested protective effect of the prime-boost regimens. Separate groups of female naïve C57BL/6 mice (5 per group) received either three weekly intra-muscular pNGVL4a-Sig/E7(detox)/HSP70 DNA vaccinations, or three intra-muscular TA-CIN protein vaccinations, or two intra-muscular pNGVL4a-Sig/E7(detox)/HSP70 DNA vaccinations followed by a single intra-muscular TA-CIN protein vaccination. Each vaccination was given on weekly intervals. One week after the last vaccination, the mice were injected subcutaneously with 2 × 10^5^ of TC-1 tumor cells (Fig. 5a). The tumor growth was monitored by palpation and caliper measurement. As shown in Fig. 5b, all of the mice vaccinated with pNGVL4a-Sig/E7(detox)/HSP70 DNA followed by TA-CIN boost were protected from TC-1 tumor growth. Two out of the five mice vaccinated with pNGVL4a-Sig/E7(detox)/HSP70 DNA three times showed initial tumor growth but the tumor regressed completely and remained tumor-free during the experimental period. In contrast, three out of the five mice vaccinated three times with TA-CIN showed initial tumor growth, and only one of these mice demonstrated complete tumor regression, while the other two died of tumor burden (Fig. 5c).

**Fig. 6:**
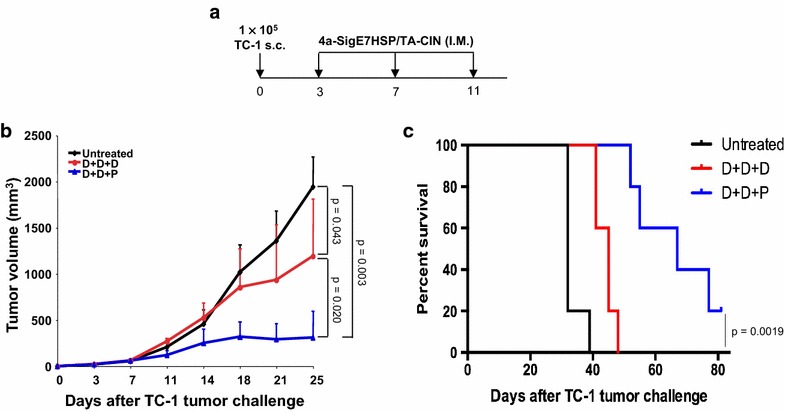
pNGVL4a-Sig/E7(detox)/HSP70 DNA priming followed by TA-CIN boost generated strong therapeutic anti-tumor effects in TC-1 tumor-bearing mice. Five to eight weeks old female C57BL/6 mice (5 mice/group) were injected with 1 × 10^5^ of TC-1 tumor cells subcutaneously. Three days later, one group of the tumor-bearing mice was vaccinated with 25 μg/mouse of pNGVL4a-Sig/E7(detox)/HSP70 DNA in 50 μl three times. Another group of mice was vaccinated with 25 μg/mouse of pNGVL4a-Sig/E7(detox)/HSP70 DNA in 50 μl twice followed by single 25 μg/mouse of TA-CIN protein in 20 μl. All vaccinations were given via intramuscular injection (leg muscle) with 4-day interval. **a** Schematic illustration of the experiment. **b** Summary of tumor volume. **c** Kaplan–Meier survival analysis of TC-1 tumor-bearing mice

To further examine the therapeutic anti-tumor immunity generated by the vaccination regimens, we challenged the mice subcutaneously (5 per group) with 1 × 10^5^ TC-1 cells. Three and 7 days after tumor challenge, mice were vaccinated intra-muscularly with 25 μg pNGVL4a-Sig/E7(detox)/HSP70 DNA. 4 days after the second vaccination, the mice were vaccinated intra-muscularly with either pNGVL4a-Sig/E7(detox)/HSP70 DNA or TA-CIN protein (Fig. 6a). Tumor size was measured with a digital caliper. As shown in Fig. 6b and c, mice vaccinated with three pNGVL4a-Sig/E7(detox)/HSP70 DNA injections generated a certain degree of anti-tumor effect, a slight reduction in tumor growth, and longer survival compared to untreated mice. However, mice vaccinated twice with pNGVL4a-Sig/E7(detox)/HSP70 DNA followed by one TA-CIN protein vaccination boost generated a significantly more potent anti-tumor effect and survived significantly longer when compared to either untreated mice or mice vaccinated with pNGVL4a-Sig/E7(detox)/HSP70 DNA vaccine only (Fig. 6b, c). These therapeutic antitumor immunity data are consistent with both the prophylactic vaccination study (Fig. 5) and the induction of HPV16 E7-specific CD8+ T cell responses in naïve mice (Fig. 3).

### Two pNGVL4a-Sig/E7(detox)/HSP70 DNA priming vaccinations followed by a single boost with TA-CIN protein is well tolerated

Previous early phase clinical trials suggest that three monthly intra-muscular vaccinations with either the TA-CIN protein (up to 533 µg/dose) [15] or the pNGVL4a-Sig/E7(detox)/HSP70 DNA (up to 3 mg/dose) [7] are well tolerated. However it is unclear whether the combination of these two vaccines will be similarly well tolerated. To address this issue, female naïve C57BL/6 mice (5 per group) were vaccinated twice with pNGVL4a-Sig/E7(detox)/HSP70 DNA and then once with TA-CIN protein, or three times with only pNGVL4a-Sig/E7(detox)/HSP70 DNA, or three times with only TA-CIN protein. As in the above clinical studies, each vaccine was administered to the mice via intramuscular injection upon a 1-month interval. The health of the mice was monitored by the measurement of injection site irritation and body weight before and after booster immunization. All vaccinated mice appeared healthy during the whole experiment period. Necropsy was performed 16 days after the last vaccination and complete blood count, clinical chemistry analyses and histopathology studies were performed for the group of mice that received three immunizations with TA-CIN, and the group of mice that received a single TA-CIN protein vaccination after two pNGVL4a-Sig/E7(detox)/HSP70 DNA vaccinations. As shown in Additional file 5: Figure S5, TA-CIN protein vaccination after pNGVL4a-Sig/E7(detox)/HSP70 DNA vaccination did not result in significant change in the body weight of the vaccinated mice. Also, no significant irritation was observed at the injection site.

The clinical chemistry, CBC and histopathology results were generally unremarkable and similar between the TA-CIN and heterologous prime-boost groups of vaccinated mice (Additional file 6: Table S1).

In general, the white blood cell counts were similar in both vaccinated groups, but were considered to be slightly elevated compared to naïve C57BL/6 mice in our facility as well as data from the Mouse Phenome Database [20]. All spleens of the vaccinated mice were slightly larger than expected for naïve mice of this age and size, with expanded white pulp, consistent with mild lymphoid hyperplasia. Lymph nodes also have enlarged or prominent follicles. These mild changes are fairly common background findings that are considered to be consistent with the vaccinated (immune stimulated) status of the mice in this study. Kidney, liver and reproductive tract changes were minimal or mild and considered to be within the range of expected background findings for C57BL/6 mice of this age.

The similar physiological status of mice from the two treatment groups suggests that priming with pNGVL4a-Sig/E7(detox)/HSP70 DNA vaccine followed by boosting with TA-CIN protein vaccine is safe and well-tolerated in preclinical model as homologous TA-CIN vaccination regimen.

## Discussion

In a previous phase I study, healthy volunteers were vaccinated intra-muscularly with TA-CIN three times at monthly intervals. A dose escalation of 26, 128 and 533 µg of TA-CIN administered in 0.5 mL was tested and HPV-specific humoral and cellular immune responses to the lowest dose were weaker than that for the intermediate and high doses, while the responses to intermediate and high doses were similar [15]. A concentration range of 1, 5 and 25 µg of TA-CIN was tested in vaccination studies of C57BL6 mice, reflecting the practical need for a smaller (0.02 mL) injection volume and their approximately 3000-fold lower weight. Likewise, the prior published human studies of pNGVL4a-Sig/E7(detox)/HSP70 DNA used three intra-muscular doses of up to 3 mg [7], whereas here the mice were vaccinated with 25 µg doses based upon the same reasoning. Both accelerated weekly and monthly schedules were used and each appeared well tolerated. As in the human studies, three intra-muscular vaccinations of mice with either pNGVL4a-Sig/E7(detox)/HSP70 DNA or TA-CIN were well tolerated. Furthermore, two intra-muscular vaccinations of mice with pNGVL4a-Sig/E7(detox)/HSP70 DNA followed by a single dose of TA-CIN was similarly well tolerated. However, this heterologous prime-boost combination elicited a more potent HPV-specific cellular immune response in mice as compared to a homologous regimen for either pNGVL4a-Sig/E7(detox)/HSP70 DNA or TA-CIN.

Intra-dermal and -muscular administration of TA-CIN generated comparable antigen-specific CD8+ T cell responses after priming with a DNA vaccine. The intra-dermal route of administration was considered as it has been suggested that boosting at or near the lesion (i.e., within the epidermis) may be important to elicit T cells that home to the site of HPV infection. However, intra-dermal vaccination is still below the basement membrane rather than in the epidermis, which may account for the similar response as seen for intra-muscular boosting with TA-CIN. Therefore, given the technical challenges of reliable intra-dermal administration in patients (e.g., as in the Mantoux test), we chose to focus on intra-muscular delivery of TA-CIN.

Recombinant protein based booster vaccines administered after DNA vaccinations in previous clinical trials were formulated with adjuvants [10–13]. Interestingly, previous clinical trials of TA-CIN protein vaccine, and studies herein, have shown that TA-CIN is capable of eliciting significant antigen-specific T cell responses without adjuvant [16, 17]. The immunogenicity of TA-CIN may reflect the unusual nature of this antigen; i.e., TA-CIN is a filterable aggregate rather than a soluble protein and this may enhance its recognition and processing by antigen-presenting cells.

Antigens presented in protein form are usually directed towards exogenous antigen presentation/MHC-II pathway. However, the protein vaccine used in this study, TA-CIN, is in a filterable particulate form. Particulate protein has been shown to be able to enter both exogenous/MHC-II pathway as well as cross presentation/MHC-I pathway [21]. Several potential mechanisms contributing to the cross presentation of particulate proteins have been proposed in previous studies [22–24]. The immunogenicity of TA-CIN and its ability to generate antigen-specific CD8+ T cells have been demonstrated in several previous studies [15–17]. These studies, in conjunction with our data, suggest that TA-CIN, without adjuvant, can be used as potential booster vaccine to enhance the antigen-specific CD8+ T cell-mediated immune responses generated by priming DNA-based vaccinations.

Several clinical trials have tested heterologous DNA prime-protein boost vaccination regimens for multiple antigens, including antigens of HIV, malaria and influenza. Such heterologous prime-boost vaccination strategies have been well tolerated and immunogenic in healthy volunteers [10–13]. However, this has not been tested against HPV-related disease. A previous clinical trial utilized TA-CIN protein as booster vaccine following priming with an HPV recombinant vaccinia virus vaccine (TA-HPV) via scarification and was well tolerated and immunogenic. However, there was no impact on HPV disease readily distinguishable from TA-HPV or TA-CIN immunization alone in this small study [17]. Similar results were obtained when the order of vaccination was switched, i.e., TA-CIN priming and TA-HPV boost [25]. Additionally, pNGVL4a-Sig/E7(detox)/HSP70 DNA priming has been used prior to a single intra-muscular TA-HPV booster immunization and was well tolerated and immunogenic in a small phase I study (NCT00788164). Notably, this intra-muscular prime-boost combination appeared able to elicit T cell responses that traffic to HPV-associated cervical intraepithelial lesions, a subset of which regressed (although it is not clear if this was above the spontaneous rate of regression) [8]. Our current findings demonstrated that heterologous DNA-prime, protein-boost vaccination regimen is capable of eliciting a superior antigen-specific immune response compared to homologous DNA or protein vaccination in mice, while maintaining similar safety profile. These findings suggest that further study of pNGVL4a-Sig/E7(detox)/HSP70 DNA priming vaccination followed by a single TA-CIN protein boost is warranted. Compared to boosting with TA-HPV, use of TA-CIN protein lacks the potential risks associated with live vaccinia virus, especially for immunocompromised patients.

The TA-CIN boosting strategy was effective for boosting E7-specific T cell responses after several different DNA-based priming vaccines, including pNGVL4a-Sig/E7(detox)/HSP70, pNGVL4a-CRT/E7(detox) and pNGVL4a-CRT-E6E7L2, suggesting this is a general phenomenon. Interestingly, the enhancement of antigen-specific immune response of mice upon a TA-CIN boost following priming with therapeutic HPV DNA vaccine seemed to be restricted to E7-specific CD8+ T cell response, even when priming using pNGVL4a-CRT-E6E7L2 (Additional file 4: Figure S4). As shown in Additional file 2: Figure S2 and Additional file 4: Figure S4, mice primed twice with DNA followed by one TA-CIN boost at 1 week intervals generated only comparable E7-specific CD4 and E6-specific CD8+ T cell responses compared to those receiving only DNA or protein vaccinations. This likely reflects the major dominance of HPV16 E7 over E6 in C57BL6 mice, while E6 may be dominant in other backgrounds including in patients.

Of note, in this study we have shown that priming with pNGVL4a-CRT-E6E7L2 DNA vaccine followed by boosting with TA-CIN protein vaccine is capable of generating a TA-CIN-specific CD4+ T cell response. So far, no HPV16 E6-specific CD4+ T cell responses have been reported in C57BL/6 mice. We have tried to explore the generation of L2-specific CD4+ T cells in previous study [18]. In that study, we showed that mice vaccinated with TA-CIN generated very low CD4+ T cell response when pulsed with E7 protein but a significant CD4+ T cell response when stimulated by TA-CIN protein or CT26 cells transfected with DNA encoding L2 protein, suggesting that the CD4+ T cell responses generated by TA-CIN are mainly L2-specific. In the current study, we have also tested and showed that E7-specific CD4+ T cell responses generated by the tested regimens are extremely weak compared to TA-CIN-specific CD4+ T cell responses. Thus, we believe the TA-CIN-specific CD4+ T cell response is predominantly L2-specific.

TA-CIN protein vaccination also elicits antigen-specific antibody responses. As shown in Additional file 3: Figure S3B, TA-CIN boost following pNGVL4a-Sig/E7(detox)/HSP70 DNA prime in various vaccination regimens generates comparable levels of E7-specific serum antibodies that are slightly lower than the level generated by protein vaccinations alone, while DNA vaccination alone did not generate a detectable antibody response. However, three intra-muscular vaccinations with pNGVL4a-CRT-E6E7L2 did generate a weak L2-specific antibody response, and a single TA-CIN boost following two pNGVL4a-CRT-E6E7L2 immunizations generated similar levels of L2-specific antibodies compared to three immunizations with TA-CIN protein alone (Additional file 3: Figure S3D), suggesting the priming of B cell immunity by the DNA vaccination. The pNGVL4a-CRT-E6E7L2 vector expresses HPV16 L2 11–200, a polypeptide shown to provide cross-protection against divergent papillomavirus types in the rabbit challenge model. Furthermore vaccination of patients with TA-CIN has previously been shown to induce cross-neutralizing serum antibodies. These findings suggest that a single TA-CIN vaccination, with priming using pNGVL4a-CRT-E6E7L2 DNA, may elicit L2-specific cross-neutralizing antibodies that could reduce the potential for re-infection or spread in an infected host. In addition, earlier studies have also suggested the potential for L2-specific therapeutic immunity [26, 27].

## Conclusion

In summary, our results show that priming twice with a therapeutic DNA vaccine such as pNGVL4a-Sig/E7(detox)/HSP70 DNA, or pNGVL4a-CRT/E7(detox) and pNGVL4a-CRT-E6E7L2 followed by a single TA-CIN protein booster immunization generates potent systemic HPV-specific CD8+ T cell responses. This vaccination regimen can potentially be applied for prophylaxis or to treat HPV-associated diseases ranging from persistent infection to high-grade intraepithelial neoplasia and possibly even cancer. Given that both pNGVL4a-Sig/E7(detox)/HSP70 DNA and TA-CIN protein were shown to be well tolerated in a number of early phase trials in HPV+ patients, and the absence of clinically apparent side effects when used in combination in the murine naïve and TC-1 tumor bearing animal studies described herein, this regimen is also likely to be well tolerated and immunogenic in HPV+ patients and therefore warrants further exploration in HPV+ patients.

## Methods

### Mice

Five to eight weeks old female C57BL/6 mice were purchased from Charles River Laboratories (Frederick, MD). All mice were maintained at Johns Hopkins University School of Medicine (Baltimore, MD) animal facility under specific-pathogen free conditions. All procedures were performed according to protocols approved by the Johns Hopkins Institutional Animal Care and Use Committee and in accordance with recommendations for the proper use and care of laboratory animals.

### Peptides, antibodies and regents

HPV16 E6aa50–57 peptide (YDFAFRDL), HPV16 E7aa49–57 peptide (RAHYNIVTF), and HPV16 E7aa31–68 peptide were synthesized by Macromolecular Resources (Denver, CO) at a purity of ≥80 %. FITC, PE-conjugated anti-mouse CD4 (clone RM4–5) and CD8a (clone 53.6.7), FITC-conjugated anti-mouse IFN-γ (clone XMG1.2) antibodies were purchased from BD Pharmingen (San Diego, CA). PE-conjugated, HPV16 E7aa49–57 peptide loaded H-2D^b^ tetramers were obtained from the National Institute of Allergy and Infectious Diseases Tetramer Facility.

### Cell line

HPV-16 E6 and E7-expressing TC-1 cells were generated as previously described [28]. The cells were maintained in RPMI medium supplemented with 2 mM glutamine, 1 mM sodium pyruvate, 100 IU/ml penicillin, 100 μg/ml streptomycin and 10 % fetal bovine serum (FBS).

### Vaccine and vaccination

The production of TA-CIN has been described previously [15]. The generation of pNGVL4a-Sig/E7(detox)/HSP70 [6], pNGVL4a-CRT/E7(detox) [29], and pNGVL4a-CRT-E6E7L2 [30] DNA vaccines have also been described previously, but detox mutations were incorporated in the latter. For protein vaccination, TA-CIN (Batch# 0907GP) was prepared in 20 µL and injected either intradermally (lower back) or intramuscularly (biceps femoris muscle). DNA vaccines were prepared using endotoxin-free kit (Qiagen) and injected into biceps femoris muscle in 50 µL.

### Tetramer staining

For tetramer staining, PBMCs from the mice were stained with purified anti-mouse CD16/32 (Fc block, BD Pharmingen, San Diego, CA) first, and then stained with anti-mouse CD8-FITC, and PE-conjugated H-2D^b^ tetramer loaded with HPV16 E7aa49–57 peptide at 4 °C. After washing, the cells were stained with 7-AAD before flow cytometry analysis to exclude dead cells. The cells were acquired with FACSCalibur flow cytometer and analyzed with CellQuest software.

### Intracellular cytokine staining and flow cytometry analysis

To detect HPV16 E6 or E7-specific CD8+ T cell responses by IFN-γ intracellular staining, splenocytes were stimulated with either HPV16 E6aa50–57 or E7aa49–57 peptide (1 µg/mL) at the presence of GolgiPlug (BD Pharmingen, San Diego, CA) at 37 °C overnight. To detect HPV16 E7-specific CD4+ T cell responses, splenocytes were stimulated with 4 µg/mL of HPV16 E7aa31–68 peptide, and for the detection of TA-CIN-specific CD4+ and CD8+ T cell responses, splenocytes were stimulated with 10 µg/mL of TA-CIN protein for 24 h at 37 °C. GolgiPlug was then added to the cells and further incubated overnight at 37 °C. The stimulated splenocytes were then washed once with PBS containing 0.5 % BSA and stained with either PE-conjugated anti-mouse CD4 or CD8 antibody. Cells were permeabilized and fixed with Cytofix/Cytoperm kit according to the manufacturer’s instruction (BD Pharmingen, San Diego, CA). Intracellular IFN-γ was stained with FITC-conjugated rat antimouse IFN-γ. Flow cytometry analysis was performed using FACSCalibur flow cytometer with CellQuest software (BD biosciences, Mountain View, CA).

### Elisa

HPV16 E7-specific antibody response [31] and HPV16 L2-specific antibody response [32] was detected by an enzyme-linked immunoabsorbent assay (ELISA) as described previously. Optical density (OD) value was read with xMark Microplate Spectrophotometer (BioRad, Hercules, CA) ELISA reader at 450 nm.

### In vivo tumor protection experiment

For the in vivo tumor protection experiment, female C57BL/6 mice (five per group) was vaccinated with pNGVL4a-Sig/E7(detox)/HSP70 DNA twice and followed by single TA-CIN vaccination; another group of mice was vaccinated with pNGVL4a-Sig/E7(detox)/HSP70 DNA three times. The third group of mice was vaccinated three times with TA-CIN. All of the vaccination was given through intramuscular injection with 1-week interval. Seven days after the last vaccination, mice were challenged with subcutaneous injection of 2 × 10^5^ of TC-1 tumor cells. The growth of the tumor was monitored twice a week by palpation and digital caliper measurement. To record the survival of the tumor-bearing mice, either natural death or a tumor diameter greater than 2 cm leading to death was counted as death.

### In vivo tumor treatment experiment

For the in vivo tumor treatment experiment, female C57BL/6 mice (five per group) were injected with 1 × 10^5^ of TC-1 tumor cells subcutaneously. Three days after tumor cell injection, one group of mice was vaccinated with pNGVL4a-Sig/E7(detox)/HSP70 DNA twice and followed by a single TA-CIN vaccination. Another group of mice was vaccinated with pNGVL4a-Sig/E7(detox)/HSP70 DNA three times. All vaccinations were given through intramuscular injection with 4 days interval. The growth of the tumor was monitored twice a week by palpation and digital caliper measurement. Tumor volume was calculated using the formula [largest diameter × (perpendicular diameter) 2] × 3.14/6. To record the survival of the tumor-bearing mice, either natural death or a tumor diameter greater than 2 cm leading to death was counted as death.

### Safety assessment of pNGVL4a-Sig/E7(detox)/HSP70 DNA priming vaccinations followed by a single boost with TA-CIN protein

For the safety assessment, one group of female naïve C57BL/6 mice was vaccinated with pNGVL4a-Sig/E7(detox)/HSP70 DNA twice and followed by single TA-CIN vaccination. Another group of mice was vaccinated with pNGVL4a-Sig/E7(detox)/HSP70 DNA three times. The third group of mice was vaccinated three times with TA-CIN. All of the vaccination was given via intramuscular injection at 1-month intervals. The health of the mice was monitored by the measurement of body weight with a digital scale (Ohaus, Parsippany, NJ), food consumption and injection site irritation. Sixteen days after the last vaccination, mice were euthanized and necropsy, complete blood count (CBC), clinical chemistry and histopathology analyses were performed by Phenotyping Core, Department of Molecular and Comparative Pathobiology, the Johns Hopkins University as described previously [33], and a report was prepared by Dr. Cory Brayton.

### Statistical analysis

Data were expressed as means ± standard deviations (SD). Comparisons between individual data point was analyzed by two-tailed Student’s *t* test. A *P* value of less than 0.05 was considered statistically significant.

## Additional files

10.1186/s13578-016-0080-z Comparison of HPV16 E7-specific CD8+ T cell responses induced by TA-CIN boost at different dose and vaccination route following twice pNGVL4a-Sig/E7(detox)/HSP70 DNA prime. 5 ~ 8 weeks old female C57BL/6 mice (5 mice/group) were vaccinated twice with 25 μg/mouse of pNGVL4a-Sig/E7(detox)/HSP70 DNA in 50 μl via intramuscular injection (leg muscle), followed by i.m. vaccination of indicated dose of TA-CIN in 20 μl, at one week interval. 7 days after last vaccination, PBMCs were prepared and stained with anti-mouse CD8 and HPV16 E7 tetramer. The data were acquired with FACSCalibur and analyzed with CellQuest. A and B. Flow cytometry analysis of HPV16 E7-specific CD8+ T cells in peripheral blood.
10.1186/s13578-016-0080-z Detection of HPV16 E7-specific CD4+ T cell responses induced by TA-CIN vaccination at different dose and vaccination route when combined with pNGVL4a-Sig/E7(detox)/HSP70 DNA vaccination. Briefly, 5 ~ 8 weeks old female C57BL/6 mice (5 mice/group) were vaccinated with either 25 μg/mouse of pNGVL4a-Sig/E7(detox)/HSP70 DNA in 50 μl via intramuscular injection (leg muscle) or indicated dose of TA-CIN in 20 μl via either i.d. or i.m. injection. The mice were boosted as indicated twice with one-week interval. Two weeks after last vaccination, splenocytes were prepared and stimulated with 4 μg/ml of HPV16 E7aa31–68 peptide for 24 h and further cultured at the presence of GolgiPlug (1 μl/ml) overnight at 37 °C. The cells were then stained with anti-mouse CD4 followed by intracellular IFN-γ. The data were acquired with FACSCalibur and analyzed with CellQuest. A. Schematic illustration of the experiment. B. Flow cytometry analysis of HPV16 E7-specific CD4+ T cells in spleen. C. Summary of the flow cytometry data.
10.1186/s13578-016-0080-z Detection of HPV16 E7 and L2-specific antibody responses after vaccination by ELISA. A. Schematic illustration of the experiment for the detection of HPV 16 E7-specific antibody response. 5 ~ 8 weeks old female C57BL/6 mice (5 mice/group) were vaccinated with 25 μg/mouse of pNGVL4a-Sig/E7(detox)/HSP70 in 50 μl via intramuscular injection or 5 or 25 μg/mouse of TA-CIN in 20 μl via intradermal/intramuscular injection. The mice were boosted as indicated twice with one-week interval. 13 days after last vaccination, sera were prepared from the mouse and stored at −20 °C until analysis. B. HPV16 E7-specific antibody response after vaccination. C. Schematic illustration of the experiment for the detection of HPV 16 L2-specific antibody response. 5 ~ 8 weeks old female C57BL/6 mice (5 mice/group) were vaccinated with 5 μg/mouse of pNGVL4a-CRT-E6E7L2 DNA in 50 μl via intramuscular injection or 25 μg/mouse of TA-CIN in 20 μl via intramuscular injection. The mice were boosted as indicated twice with one-week interval. 13 days after last vaccination, sera were prepared from the mouse and stored at -20 °C until analysis. D. HPV16 L2-specific antibody response after vaccination.
10.1186/s13578-016-0080-z Detection of HPV16 E6-specific CD8+ T cell and TACIN-specific CD4+ T cell responses induced by intradermal TA-CIN vaccination and intramuscular pNGVL4a-CRT-E6E7L2 DNA vaccination. Briefly, 5 ~ 8 weeks old female C57BL/6 mice (5 mice/group) were vaccinated with either 5 μg/mouse of pNGVL4a-CRT-E6E7L2 DNA in 50 μl via intramuscular injection (leg muscle) or 25 μg/mouse of TA-CIN in 20 μl via intradermal injection. The mice were boosted as indicated twice with one-week interval. Two weeks after last vaccination, splenocytes were prepared and stimulated with 20 μg/ml of TA-CIN protein for 24 h and further cultured at the presence of GolgiPlug (1 μl/ml) overnight at 37 °C and stained with anti-mouse CD4 followed by intracellular IFN-γ staining. The splenocytes were alternatively stimulated with 1 μg/ml of HPV16 E6aa50–57 peptide at the presence of GolgiPlug (1 μl/ml) overnight at 37 °C. The cells were then stained with anti-mouse CD8 followed by intracellular IFN-γ staining. The data were acquired with FACSCalibur and analyzed with CellQuest. A. Schematic illustration of the experiment. B and C. Flow cytometry analysis of TACIN-specific CD4+ T cells in spleen. D and E. Flow cytometry analysis of HPV16 E6-specific CD8+ T cells in spleen.
10.1186/s13578-016-0080-z No detection of significant change in body weight of vaccinated mice. 5 ~ 8 weeks old female C57BL/6 mice (5 mice/group) was vaccinated with 25 μg/mouse of pNGVL4a-Sig/E7(detox)/HSP70 DNA in 50 μl three times (A). Another group of mice was vaccinated with 25 μg/mouse of TA-CIN protein in 20 μl three times (B). The third group of mice was vaccinated with 25 μg/mouse of pNGVL4a-Sig/E7(detox)/HSP70 DNA in 50 μl twice followed by single 25 μg/mouse of TA-CIN protein in 20 μl (C). All vaccinations were given via intramuscular injection (leg muscle) with 1-month interval. The body weight was measured with a digital scale.
10.1186/s13578-016-0080-z Pathology Report of Mice Receiving Three Protein Vaccination (PPP) or Heterologous Prime-Boost Vaccination (DDP).

## Abbreviations

HPV: human papillomavirus
MHC: major histocompatibility complex
IFN: interferon
VIN: vulvar intraepithelial neoplasia
VaIN: vaginal intraepithelial neoplasia
HIV: human immunodeficiency virus
PE: phycoerythrin
FITC: fluorescein isothlocyanate
