# Supplementary figures and images for: Optimization of heterologous DNA-prime, protein boost regimens and site of vaccination to enhance therapeutic immunity against human papillomavirus-associated disease
Source: Cell Biosci. 2016 Feb 25;6:16. doi: 10.1186/s13578-016-0080-z (PMC4766698; doi:10.1186/s13578-016-0080-z)

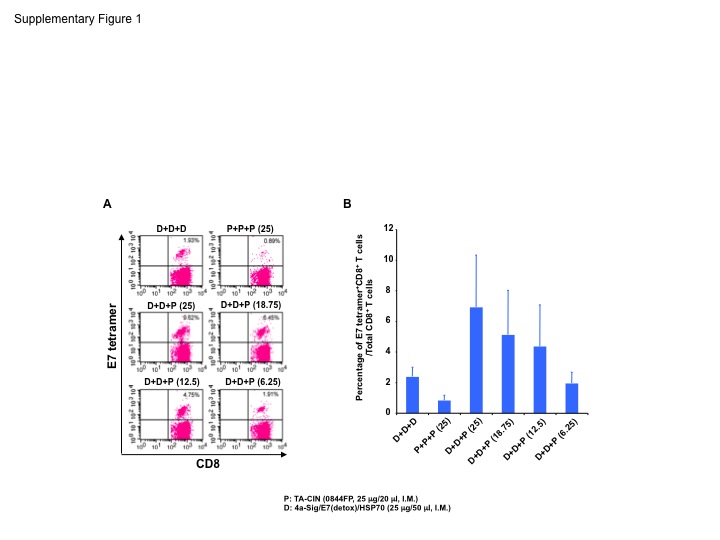

Supplement: Supplementary file 1 — 10.1186/s13578-016-0080-z Comparison of HPV16 E7-specific CD8+ T cell responses induced by TA-CIN boost at different dose and vaccination route following twice pNGVL4a-Sig/E7(detox)/HSP70 DNA prime. 5 ~ 8 weeks old female C57BL/6 mice (5 mice/group) were vaccinated twice with 25 μg/mouse of pNGVL4a-Sig/E7(detox)/HSP70 DNA in 50 μl via intramuscular injection (leg muscle), followed by i.m. vaccination of indicated dose of TA-CIN in 20 μl, at one week interval. 7 days after last vaccination, PBMCs were prepared and stained with anti-mouse CD8 and HPV16 E7 tetramer. The data were acquired with FACSCalibur and analyzed with CellQuest. A and B. Flow cytometry analysis of HPV16 E7-specific CD8+ T cells in peripheral blood. [file 13578_2016_80_MOESM1_ESM.jpg]

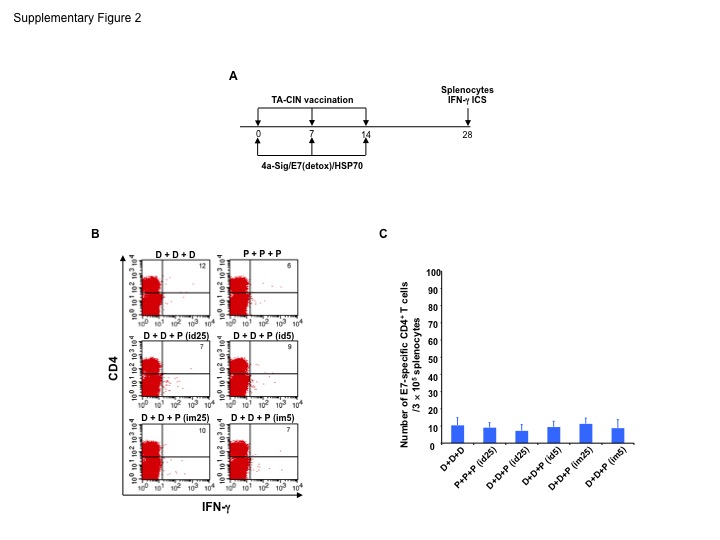

Supplement: Supplementary file 2 — 10.1186/s13578-016-0080-z Detection of HPV16 E7-specific CD4+ T cell responses induced by TA-CIN vaccination at different dose and vaccination route when combined with pNGVL4a-Sig/E7(detox)/HSP70 DNA vaccination. Briefly, 5 ~ 8 weeks old female C57BL/6 mice (5 mice/group) were vaccinated with either 25 μg/mouse of pNGVL4a-Sig/E7(detox)/HSP70 DNA in 50 μl via intramuscular injection (leg muscle) or indicated dose of TA-CIN in 20 μl via either i.d. or i.m. injection. The mice were boosted as indicated twice with one-week interval. Two weeks after last vaccination, splenocytes were prepared and stimulated with 4 μg/ml of HPV16 E7aa31–68 peptide for 24 h and further cultured at the presence of GolgiPlug (1 μl/ml) overnight at 37 °C. The cells were then stained with anti-mouse CD4 followed by intracellular IFN-γ. The data were acquired with FACSCalibur and analyzed with CellQuest. A. Schematic illustration of the experiment. B. Flow cytometry analysis of HPV16 E7-specific CD4+ T cells in spleen. C. Summary of the flow cytometry data. [file 13578_2016_80_MOESM2_ESM.jpg]

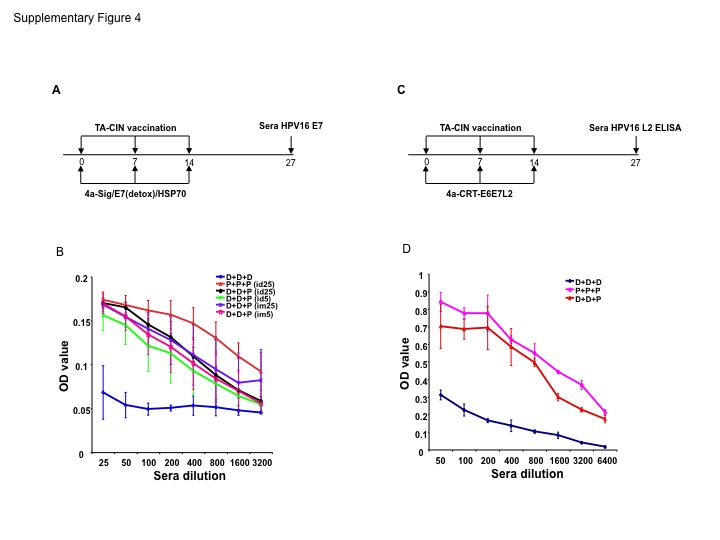

Supplement: Supplementary file 3 — 10.1186/s13578-016-0080-z Detection of HPV16 E7 and L2-specific antibody responses after vaccination by ELISA. A. Schematic illustration of the experiment for the detection of HPV 16 E7-specific antibody response. 5 ~ 8 weeks old female C57BL/6 mice (5 mice/group) were vaccinated with 25 μg/mouse of pNGVL4a-Sig/E7(detox)/HSP70 in 50 μl via intramuscular injection or 5 or 25 μg/mouse of TA-CIN in 20 μl via intradermal/intramuscular injection. The mice were boosted as indicated twice with one-week interval. 13 days after last vaccination, sera were prepared from the mouse and stored at −20 °C until analysis. B. HPV16 E7-specific antibody response after vaccination. C. Schematic illustration of the experiment for the detection of HPV 16 L2-specific antibody response. 5 ~ 8 weeks old female C57BL/6 mice (5 mice/group) were vaccinated with 5 μg/mouse of pNGVL4a-CRT-E6E7L2 DNA in 50 μl via intramuscular injection or 25 μg/mouse of TA-CIN in 20 μl via intramuscular injection. The mice were boosted as indicated twice with one-week interval. 13 days after last vaccination, sera were prepared from the mouse and stored at -20 °C until analysis. D. HPV16 L2-specific antibody response after vaccination. [file 13578_2016_80_MOESM3_ESM.jpg]

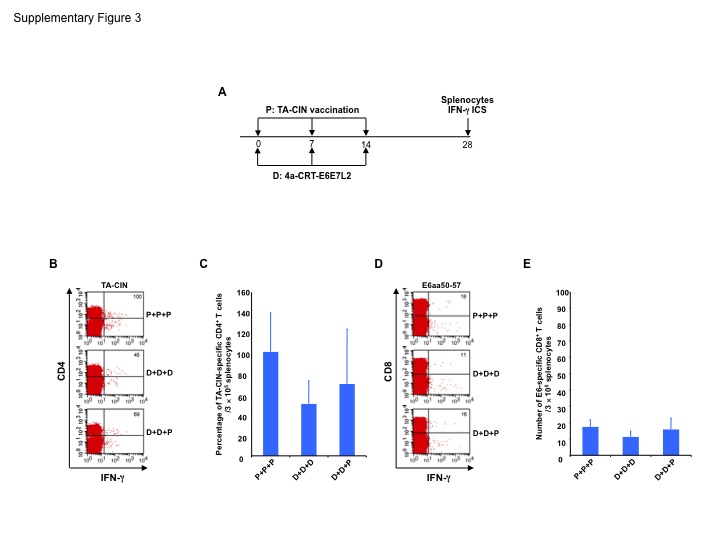

Supplement: Supplementary file 4 — 10.1186/s13578-016-0080-z Detection of HPV16 E6-specific CD8+ T cell and TACIN-specific CD4+ T cell responses induced by intradermal TA-CIN vaccination and intramuscular pNGVL4a-CRT-E6E7L2 DNA vaccination. Briefly, 5 ~ 8 weeks old female C57BL/6 mice (5 mice/group) were vaccinated with either 5 μg/mouse of pNGVL4a-CRT-E6E7L2 DNA in 50 μl via intramuscular injection (leg muscle) or 25 μg/mouse of TA-CIN in 20 μl via intradermal injection. The mice were boosted as indicated twice with one-week interval. Two weeks after last vaccination, splenocytes were prepared and stimulated with 20 μg/ml of TA-CIN protein for 24 h and further cultured at the presence of GolgiPlug (1 μl/ml) overnight at 37 °C and stained with anti-mouse CD4 followed by intracellular IFN-γ staining. The splenocytes were alternatively stimulated with 1 μg/ml of HPV16 E6aa50–57 peptide at the presence of GolgiPlug (1 μl/ml) overnight at 37 °C. The cells were then stained with anti-mouse CD8 followed by intracellular IFN-γ staining. The data were acquired with FACSCalibur and analyzed with CellQuest. A. Schematic illustration of the experiment. B and C. Flow cytometry analysis of TACIN-specific CD4+ T cells in spleen. D and E. Flow cytometry analysis of HPV16 E6-specific CD8+ T cells in spleen. [file 13578_2016_80_MOESM4_ESM.jpg]

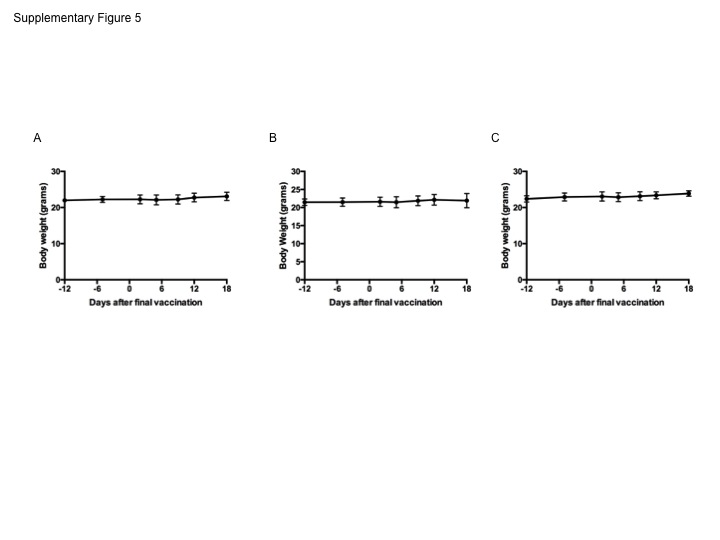

Supplement: Supplementary file 5 — 10.1186/s13578-016-0080-z No detection of significant change in body weight of vaccinated mice. 5 ~ 8 weeks old female C57BL/6 mice (5 mice/group) was vaccinated with 25 μg/mouse of pNGVL4a-Sig/E7(detox)/HSP70 DNA in 50 μl three times (A). Another group of mice was vaccinated with 25 μg/mouse of TA-CIN protein in 20 μl three times (B). The third group of mice was vaccinated with 25 μg/mouse of pNGVL4a-Sig/E7(detox)/HSP70 DNA in 50 μl twice followed by single 25 μg/mouse of TA-CIN protein in 20 μl (C). All vaccinations were given via intramuscular injection (leg muscle) with 1-month interval. The body weight was measured with a digital scale. [file 13578_2016_80_MOESM5_ESM.jpg]
