# Supplementary material for: Optimization of heterologous DNA-prime, protein boost regimens and site of vaccination to enhance therapeutic immunity against human papillomavirus-associated disease
Source: Cell Biosci. 2016 Feb 25;6:16. doi: 10.1186/s13578-016-0080-z (PMC4766698; doi:10.1186/s13578-016-0080-z)
Supplement: Supplementary file 6 — 10.1186/s13578-016-0080-z Pathology Report of Mice Receiving Three Protein Vaccination (PPP) or Heterologous Prime-Boost Vaccination (DDP). [file 13578_2016_80_MOESM6_ESM.docx]

| **MCP** | **67860** | **67861** | **67862** | **67863** | **67864** | **67865** | **67866** | **67867** | **67868** | **67869** |
| --- | --- | --- | --- | --- | --- | --- | --- | --- | --- | --- |
| **RED** | 7/28/15 | 7/28/15 | 7/28/15 | 7/28/15 | 7/28/15 | 7/28/15 | 7/28/15 | 7/28/15 | 7/28/15 | 7/28/15 |
| **PI** | Wu, TC | Wu, TC | Wu, TC | Wu, TC | Wu, TC | Wu, TC | Wu, TC | Wu, TC | Wu, TC | Wu, TC |
| **Species** | Mouse | Mouse | Mouse | Mouse | Mouse | Mouse | Mouse | Mouse | Mouse | Mouse |
| **Strain** | C57BL/6 | C57BL/6 | C57BL/6 | C57BL/6 | C57BL/6 | C57BL/6 | C57BL/6 | C57BL/6 | C57BL/6 | C57BL/6 |
| **Age (Week)** | 20 | 20 | 20 | 20 | 20 | 20 | 20 | 20 | 20 | 20 |
| **Sex** | Female | Female | Female | Female | Female | Female | Female | Female | Female | Female |
| **ID** | PPP-2 | PPP-3 | PPP-4 | PPP-1 | PPP-5 | DDP-5 | DDP-1 | DDP-3 | DDP-2 | DDP-4 |
| **History + request** | 8-10 week old vaccinated 25μg TA-CIN IM R biceps femoris. Boost x 2 | 8-10 week old vaccinated 25μg TA-CIN IM R biceps femoris. Boost x 2 | 8-10 week old vaccinated 25μg TA-CIN IM R biceps femoris. Boost x 2 | 8-10 week old vaccinated 25μg TA-CIN IM R biceps femoris. Boost x 2 | 8-10 week old vaccinated 25μg TA-CIN IM R biceps femoris. Boost x 2 | 8-10 week old vaccinated 25μg 4a-sig E7(detox)HSP IM R biceps femoris twice + 25 μg TA-CIN IM R biceps femoris once | 8-10 week old vaccinated 25μg 4a-sig E7(detox)HSP IM R biceps femoris twice + 25 μg TA-CIN IM R biceps femoris once | 8-10 week old vaccinated 25μg 4a-sig E7(detox)HSP IM R biceps femoris twice + 25 μg TA-CIN IM R biceps femoris once | 8-10 week old vaccinated 25μg 4a-sig E7(detox)HSP IM R biceps femoris twice + 25 μg TA-CIN IM R biceps femoris once | 8-10 week old vaccinated 25μg 4a-sig E7(detox)HSP IM R biceps femoris twice + 25 μg TA-CIN IM R biceps femoris once |
| **Gross** | Alive 5 months female black mouse; subjective BCS 3/5; WNL | Alive 5 months female black mouse; subjective BCS 3/5; WNL | Alive 5 months female black mouse; subjective BCS 3/5; WNL | Alive 5 months female black mouse; subjective BCS 3/5; WNL | Alive 5 months female black mouse; subjective BCS 3/5; WNL | Alive 5 months female black mouse; subjective BCS 3/5; WNL | Alive 5 months female black mouse; subjective BCS 3/5; WNL | Alive 5 months female black mouse; subjective BCS 3/5; WNL | Alive 5 months female black mouse; subjective BCS 3/5; WNL | Alive 5 months female black mouse; subjective BCS 3/5; WNL |
| **Cardiocentesis** | ~600μl | ~600μl | ~500μl | ~600μl | ~600μl | ~600μl | ~600μl | ~600μl | ~600μl | ~700μl |
| **Chemistry** | | | | | | | | | | |
| **CHOL** | 98 | 79 | 89 | 78 | 83 | 93 | 101 | 103 | 85 | 106 |
| **HDL** | 49 | 39 | 47 | 38 | 40 | 46 | 43 | 48 | 41 | 49 |
| **nonHDL** | 28 | 26 | 20 | 29 | 29 | 27 | 39 | 39 | 23 | 37 |
| **TRIG** | 105 | 71 | 112 | 56 | 71 | 99 | 95 | 82 | 105 | 100 |
| **UA** | 2.1 | 3.1 | 2.7 | 2.3 | 2.5 | 3.5 | 2.9 | 3.4 | 2.9 | 2.6 |
| **CK** | 342 | 297 | 227 | 204 | 128 | 161 | 240 | 116 | 85 | 49 |
| **GGT** | 3 | 3 | 6 | 4 | 2 | 5 | 3 | 7 | 4 | 5 |
| **ALT** | 25 | 24 | 31 | 19 | 24 | 37 | 24 | 40 | 31 | 15 |
| **AST** | 98 | 110 | 69 | 70 | 72 | 73 | 86 | 72 | 67 | 52 |
| **AMYL** | 843 | 1345 | 795 | 751 | 816 | 905 | 1222 | 735 | 832 | 859 |
| **LDH** | 276 | 223 | 298 | 253 | 198 | 188 | 175 | 219 | 220 | 137 |
| **ALP** | 116 | 111 | 109 | 102 | 87 | 94 | 98 | 131 | 118 | 105 |
| **TBILI** | 0.2 | 0.2 | 0.2 | 0.2 | 0.2 | 0.2 | 0.2 | 0.2 | 0.2 | 0.2 |
| **GLU** | 191 | 375 | 244 | 292 | 244 | 303 | 299 | 380 | 300 | 317 |
| **TPROT** | 5.4 | 5.6 | 5.6 | 5.1 | 5.5 | 5.4 | 5.5 | 5.7 | 5.9 | 5.6 |
| **MG** | 4.3 | 3.4 | 3.3 | 3.3 | 3.6 | 5.4 | 5.5 | 5.7 | 5.9 | 5.6 |
| **CA** | 9.8 | 10.9 | 10.4 | 10.3 | 10.6 | 9.9 | 10.4 | 10.9 | 10.4 | 10.5 |
| **BUN** | 23 | 21 | 25 | 23 | 18 | 17 | 16 | 17 | 22 | 15 |
| **CREAT** | 0.3 | 0.3 | 0.3 | 0.4 | 0.2 | 0.2 | 0.3 | 0.3 | 0.3 | 0.2 |
| **DBILI** | 0.2 | 0.2 | 0.2 | 0.1 | 0.2 | 0.1 | 0.1 | 0.1 | 0.2 | 0.2 |
| **ALB** | 3.3 | 3.4 | 3.2 | 3.1 | 3.4 | 3.1 | 3.2 | 3.4 | 3.4 | 3.3 |
| **CBC** | | | | | | | | | | |
| **WBC (K/μL)** | 7.76 | 6.38 | 5.86 | 4.44 | 7.13 | 8.03 | 7.35 | 7.32 | 4.85 | 5.21 |
| **NE (K/μL)** | 0.71 | 0.83 | 0.65 | 0.85 | 0.76 | 0.65 | 0.56 | 0.71 | 0.49 | 0.42 |
| **LY (K/μL)** | 5.67 | 4.82 | 4.91 | 3.14 | 4.38 | 6.62 | 6.39 | 5.86 | 4.11 | 4.6 |
| **MO (K/μL)** | 1.2 | 0.56 | 0.14 | 0.34 | 1.86 | 0.67 | 0.27 | 0.7 | 0.21 | 0.11 |
| **RBC (M/μL)** | 9.87 | 9.59 | 9.91 | 9.4 | 9.29 | 9.69 | 9.19 | 9.61 | 10.01 | 9.71 |
| **Hb (g/dL)** | 14.1 | 13.8 | 14.5 | 14 | 13.6 | 14.2 | 13 | 14 | 14.3 | 13.7 |
| **HCT (%)** | 48.7 | 46.1 | 49.3 | 45.7 | 43.6 | 46.1 | 44.5 | 47.3 | 49.3 | 47.1 |
| **PLT (K/μL)** | 906 | 1068 | 1001 | 1100 | 941 | 1165 | 990 | 1009 | 1086 | 1032 |

Additional file 6: Table S1. Pathology Report of Mice Receiving Three Protein Vaccination (PPP) or Heterologous Prime-Boost Vaccination (DDP)

| **MCP** | **67860** | **67861** | **67862** | **67863** | **67864** | **67865** | **67866** | **67867** | **67868** | **67869** |
| --- | --- | --- | --- | --- | --- | --- | --- | --- | --- | --- |
| **Body Weight** | 23.901 | 23.700 | 20.250 | 19.338 | 20.834 | 26.859 | 23.104 | 22.58 | 21.48 | 24.405 |
| **Organ Weights (g) perfused** | | | | | | | | | | |
| **Liver** | 1.585 | 1.546 | 1.210 | 1.356 | 1.29 | 1.623 | 1.496 | 1.432 | 1.365 | 1.513 |
| **Spleen** | 0.100 | 0.128 | 0.090 | 0.096 | 0.08 | 0.099 | 0.097 | 0.102 | 0.096 | 0.103 |
| **Heart** | 0.177 | 0.229 | 0.150 | 0.146 | 0.172 | 0.234 | 0.162 | 0.155 | 0.154 | 0.151 |
| **Right Kidney** | 0.187 | 0.212 | 0.160 | 0.161 | 0.183 | 0.2 | 0.189 | 0.192 | 0.168 | 0.206 |
| **Left Kidney** | 0.169 | 0.207 | 0.150 | 0.163 | 0.176 | 0.201 | 0.172 | 0.176 | 0.18 | 0.187 |
| **Histology** | | | | | | | | | | |
| **Slide 1: Heart Etc** | | | | | | | | | | |
| **Heart** | WNL cw perfusion | Inflam<1 +PMN heart base | WNL Hemorrhage <1 cw perimortem | WNL cw perfusion | WNL cw perfusion | WNL cw perfusion | WNL, QNS: cw perfusion | WNL cw perfusion | WNL cw perfusion | WNL cw perfusion |
| **Sternum** | WNL | WNL | WNL | WNL | WNL | WNL | WNL | WNL | WNL | WNL |
| **Marrow** | WNL mod-hi cellularity, trilineage, + pigment + crystals | WNL mod-hi cellularity, trilineage, + pigment + crystals | WNL mod-hi cellularity, trilineage, + pigment + crystals | WNL mod-hi cellularity, trilineage, + pigment + crystals | WNL mod-hi cellularity, trilineage, + pigment + crystals | WNL mod-hi cellularity, trilineage, + pigment + crystals | WNL mod-hi cellularity, trilineage, + pigment + crystals | WNL mod-hi cellularity, trilineage, + pigment + crystals | WNL mod-hi cellularity, trilineage, + pigment + crystals | WNL mod-hi cellularity, trilineage, + pigment + crystals |
| **M:E** | 2:1 | 2:1 | 2:1 | 2:1 | 2:1 | 2:1 | 1.5:1 | 2:1 | 2:1 | 2:1 |
| **Thymus** | WNL: Distinct C/M | WNL: Distinct C/M | NT | NT | WNL: Distinct C/M | WNL: Distinct C/M | WNL: Distinct C/M | WNL: Distinct C/M | WNL: Distinct C/M | WNL: Distinct C/M |
| **Lobes/size** | 2 lobes <5mm | 2 lobes <5mm | NT | NT | 2 lobes <4mm | 2 lobes <4mm | 2 lobes <4mm | 2 lobes <4mm | 2 lobes <4mm | 2 lobes <3mm |
| **Tongue** | WNL, QNS | WNL | Inflam <1 | WNL | WNL | WNL | NT | WNL | WNL | WNL |
| **Muscle** | WNL | WNL | WNL | WNL | WNL | WNL | WNL | WNL | WNL | WNL |
| **Fat** | WNL | WNL | WNL | WNL | WNL | WNL | WNL | WNL | WNL | WNL |
| **Slide 2: Lung etc** | | | | | | | | | | |
| **Lungs** | WNL, cw perfusion/ cardiocentesis | Infilt pleural / subpleural <1 | WNL, cw perfusion/ cardiocentesis | Inflam+1+PMN, cw perfusion / cardiocentesis | WNL, hemorrhage CW cardiocentesis | Inflam <1 | WNL, cw perfusion/ cardiocentesis | WNL | Infilt <1; Mediastinal lymphoid infilt | WNL, cw perfusion/ cardiocentesis |
| **Trachea** | NT | WNL, QNS | WNL | WNL | WNL | WNL | WNL | WNL | WNL | WNL, QNS |
| **Larynx** | WNL, QNS | WNL, QNS | WNL, QNS | WNL, inflame <1 + mast submucosa | WNL | WNL | WNL, QNS | WNL, QNS | NT | NT |
| **Thyroid** | WNL, Variatn follicle size +1 | WNL, Variatn follicle size +1 | NT | WNL, Variatn follicle size +1 | WNL, Variatn follicle size +1 | WNL, 2x | WNL, Variatn follicle size +1 | WNL, Variatn follicle size +1 | WNL QNS, Variatn follicle size +1 | WNL, Variatn follicle size +1 |
| **Parathyroid** | NT | NT | NT | NT | NT | WNL | NT | NT | NT | WNL |
| **Esophagus** | WNL | WNL | WNL | WNL, QNS | WNL | WNL | WNL, QNS | WNL, QNS | WNL, QNS | WNL |
| **Aorta** | WNL | WNL | NT | NT | NT | NT | QNS | WNL, QNS | NT | WNL, QNS |
| **Fat** | WNL | WNL | WNL | WNL | WNL | WNL | WNL | WNL | WNL | WNL |
| **Other** |  |  | LN QNS – pigmented macrophages + | LN pigmented macrophages; mast cells + |  |  | Siderophages in lymphoid nodules <1mm | Mediastinum lymphoid infilt +1 |  |  |
| **Slide 3: Kidney etc** | | | | | | | | | | |
| **Kidney L** | QNS – WNL on recut | WNL | QNS proteinosis <1 | WNL | WNL | WNL Degen foci <1 | QNS – WNL on recut | QNS – WNL on recut | WNL | QNS |
| **Kidney R** | QNS – WNL on recut | Inflam <1 focal | QNS – WNL on recut | WNL | WNL | WNL | Inflam <1 focal | Inflam <1 focal | WNL | WNL |
| **Adrenal** | WNL, SCH <1 | WNL, QNS | WNL, QNS | WNL, QNS | WNL, QNS, SCH <1 | WNL, QNS, x 2 | QNS | WNL, QNS | SCH +1; pigment +1; cort nodule (<1mm) | SCH + 1; pigment + 1 |

| **MCP** | **67860** | **67861** | **67862** | **67863** | **67864** | **67865** | **67866** | **67867** | **67868** | **67869** |
| --- | --- | --- | --- | --- | --- | --- | --- | --- | --- | --- |
| **Slide 4: Salivary GI etc** | | | | | | | | | | |
| **Submandibular** | WNL, CW female | WNL, CW female | CW female, inflam +1 | WNL, CW female | WNL, CW female | WNL, CW female | WNL, CW female, inflam (<1) | WNL, CW female | WNL, CW female | WNL, CW female |
| **Sublingual** | WNL | WNL | WNL | WNL | WNL | WNL | WNL | WNL | WNL | WNL |
| **Parotid** | WNL | WNL | WNL | WNL | WNL | WNL | WNL | WNL | WNL | WNL |
| **Exorbital Lacrimal** | WNL vacuole CW lipid <1 | WNL, QNS | WNL, QNS | WNL | Inflam +1 fpcal | WNL | WNL | WNL | WNL, QNS | Inflam <1 |
| **Mammary** | NT | WNL, QNS | WNL, QNS | WNL, QNS | WNL, QNS | WNL, QNS | WNL, QNS | WNL, QNS | WNL, QNS | WNL, QNS |
| **Lymph Nodes** | WNL, 1-2mm + siderophages | 1-3mm, hyperplasia mild <1 + siderophages | WNL, <2mm, hyperplasia <1, siderophages + | WNL, <2mm, hyperplasia <1, sinus histiocytosis <1 | <2mm, hyperplasia <1 | <2mm, hyperplasia <1 | 1~3mm, hyperplasia (+1) + siderophages | <2mm, siderophages + | WNL, QNS: <2mm, hyperplasia <1, siderophages + | WNL, <2mm, hyperplasia +1, siderophages + |
| **Slide 5: Pancreas etc** | | | | | | | | | | |
| **Pancreas – Exo** | WNL | WNL | Inflam+1 + microgranuloma siderophages | Inflam<1 | WNL | WNL | Inflam<1 | WNL | Inflam<1 | WNL |
| **Pancreas – Endo** | WNL CW perfusion | WNL CW perfusion Inflam<1 perivasc | WNL CW perfusion | WNL CW perfusion | WNL CW perfusion | WNL CW perfusion | WNL CW perfusion | WNL CW perfusion | vacuolatn cytoplasm<1 | vacuolatn cytoplasm<1 |
| **Lymph Nodes** | <2mmW mild hyperplasia<1 | WNL, <2mm w | WNL, <2mm, hyperplasia <1, siderophages+ | WNL <2mm, hyperplasia <1 apop<1 | WNL <2mm, hyperplasia <1 | <3mm hyperplasia + | WNL, <2mm | WNL, 3~4mm w | WNL: <2mm hyperplasia<1 | WNL: <2mm hyperplasia<1 |
| **Mesentery / vasc** | WNL | NSF | NSF | Inflam<1 | WNL | Infilt <1 | WNL | INFLAM ++1 necrot +PMN perivasc | WNL | WNL |
| **Fat** | WNL, Adequate | WNL, Adequate | WNL, Adequate | WNL, Adequate | WNL, Adequate | WNL, Adequate | WNL, Adequate | WNL, Adequate | WNL, Adequate | WNL, Adequate |
| **Slide 6: GIT** | | | | | | | | | | |
| **Forestomach:** | WNL | WNL | QNS - recut WNL | WNL | WNL | WNL, QNS | WNL | WNL | QNS | QNS |
| **Stomach:** | WNL | WNL, QNS | NT | WNL | Microgran +1, <1mm, +hair | WNL | Inflam+1 +PMN | WNL | WNL | Inflam+1 +PMN |
| **Small Int:** | WNL | Inflam+1 +PMN | WNL | inflam <1 | Inflam+1 +PMN (+papilla dilat) | Inflam+1 +PMN | Inflam+1 +PMN | Inflam+1 +PMN | inflam <1 | Inflam+1 +PMN |
| **Cecum:** | WNL | NSF | WNL | WNL | WNL | WNL | WNL | WNL | WNL | WNL |
| **Colon:** | WNL | NSF | WNL | WNL | WNL | WNL | WNL | WNL | WNL | Inflam+1 +PMN |
| **Content:** | WNL | WNL | WNL | WNL | WNL | WNL | WNL | WNL | WNL | WNL |
| **Slide 7: LIV spleen** | | | | | | | | | | |
| **Liver:** | Inflam<1 | Inflam+1 | Inflam+1 +PMN +nec | Inflam+1 +PMN +nec | Inflam +1, +PMN, +nec | Inflam +1, +PMN, +nec | Inflam +1; +PMN | Inflam +1, +PMN, +nec | Inflam<1 | Inflam +1, +PMN, +nec |
| **Hepatocytes** | aniso<1; vacuol <1 | aniso+1; vacuol <1 | aniso<1; vacuol <1 | aniso+1; vacuol <1 | aniso<1; vacuol <1 | aniso<1; vacuol <1 | aniso+1; vacuol <1 | aniso<1; vacuol <1 | aniso+1; vacuol <1 | aniso+1; vacuol <1 |
| **Gall Bladder** | WNL | Inflam +1 + PMN | QNS - recut NT | Inflam +1 + PMN | WNL | Inflam +1, +PMN | NT | NT | NT | NT |
| **Spleen:** | Splenomegaly <2x; pigment+ | Splenomegaly <2x; pigment+ | Splenomegaly <2x; pigment+ | Splenomegaly <2x; pigment+ | Splenomegaly <2x; pigment+ | Splenomegaly <2x; pigment+ | Splenomegaly <2x; pigment+ | Splenomegaly <2x; pigment+ | Splenomegaly <2x; pigment+ | Splenomegaly <2x; pigment+ |
| **Red: White~** | Red ~<W; Ly hyperplasia ~<1 | Red ~<W; Ly hyperplasia ~<1 | Red ~<W; Ly hyperplasia ~<1 | Red ~<W; Ly hyperplasia ~<1 | Red ~<W; Ly hyperplasia ~<1 | Red ~<W; Ly hyperplasia ~<1 | Red ~<W; Ly hyperplasia ~<1 | Red ~<W; Ly hyperplasia ~<1 | Red ~<W; Ly hyperplasia ~<1 | Red ~<W; Ly hyperplasia ~<1 |

| **MCP** | **67860** | **67861** | **67862** | **67863** | **67864** | **67865** | **67866** | **67867** | **67868** | **67869** |
| --- | --- | --- | --- | --- | --- | --- | --- | --- | --- | --- |
| **Slide 8: Repro** | | | | | | | | | | |
| **Ovaries:** | WNL <2mm+ CL+fol | WNL 2-3mm+ CL+fol | WNL 2-3mm+ CL+fol | WNL <2mm+ CL+fol | WNL, x2, <2mm, +fol, +CL | WNL, x2, <2mm, +fol, +CL | WNL 2-3mm+ CL+fol | WNL 3-4mm+ CL+fol | WNL <2mm+ CL+fol | WNL 2-3mm+ CL+fol |
| **oviduct:** | WNL | WNL | WNL |  | WNL | WNL | WNL | WNL | WNL | WNL |
| **Uterus:** | WNL, <2mm w | WNL <2mm wide, cw perfusion | Myxedema / hydro/mucometra < 3mm, angiectasis cw perfusion | WNL, < 2mm w, inflam+2+PMN, CW perfusion | WNL <2mm | WNL, <2mm, dilatation <1 | WNL <2mm  hyperplasia gland(<1) | WNL, QNS <2mm w, CW perfusion | WNL <2mm W, cw perfusion Gland hyperplasia(<1) | WNL 2-3mm W, dilat<1, CW perfusion |
| **Vagina:** | WNL | WNL, QNS | Myxedema, mild | WNL PMN + | Hyperkeratosis, ++PMN exudate vs cycle | PMN ++, exudate vs cycle | WNL: Hyperkeratosis cw cycle | WNL: Hyperkeratosis cw cycle | WNL:PMNs cw cycle | WNL: Hyperkeratosis cw cycle |
| **Urin Bladder:** | WNL | WNL | NT | WNL | WNL | NT | WNL, QNS | QNS | NT | WNL |
| **Other:** | Lnode WNL <<2mm | Lnode WNL <<2mm | clitoral gland QNS WNL | Lnode < 2mm, hyperplasia <1;mammary QNS WNL |  |  |  |  |  |  |
| **Slide 9: Skin / Leg** | | | | | | | | | | |
| **Skin:** | WNL Pigmented anagen to telogen | WNL Pigmented anagen to telogen | WNL Pigmented primarily telogen | WNL Pigmented anagen to telogen | WNL, anagen and telogen | WNL, telogen >anagen | WNL Pigmented anagen to telogen | WNL Pigmented anagen to telogen | WNL: mostly telogen | WNL Pigmented anagen to telogen |
| **Inflam:** | WNL <1 | WNL <1 | WNL <1 | WNL <1 | WNL <1 | WNL <1 | WNL <1 | WNL <1 | WNL <1 | WNL <1 |
| **SC subcutis:** | WNL + fat | WNL + fat | WNL + fat | WNL + fat | WNL | WNL | WNL + fat | WNL | WNL | WNL |
| **Mammary Gl:** | WNL, QNS | WNL | WNL | WNL | WNL, QNS | WNL, QNS | WNL, QNS | WNL QNS | WNL QNS | WNL QNS |
| **LEG DECAL** | WNL, QNS | WNL, QNS | WNL, QNS | WNL, QNS | WNL, QNS | WNL, QNS | QNS | WNL, QNS | WNL, QNS | WNL, QNS |
| **Muscle** | WNL, QNS | WNL, QNS | WNL, QNS | WNL, QNS | WNL, QNS | WNL, QNS | QNS | WNL, QNS | WNL, QNS | WNL, QNS |
| **Bone** | WNL, QNS | WNL, QNS | WNL, QNS | WNL, QNS | WNL, QNS | WNL, QNS | QNS | WNL, QNS | WNL, QNS | WNL, QNS |
| **Marrow:** | WNL, QNS | WNL, QNS | WNL, QNS | WNL, QNS | WNL, QNS | WNL, QNS | QNS | CW slide1 | CW slide1 | WNL, QNS |
| **Other:** | clitoral gland WNL |  | mammary QNS WNL |  |  |  |  |  |  |  |
| **Slide 10: Head Decal** | | | | | | | | | | |
| **Cerebrum:** | WNL | WNL | WNL | WNL | WNL, QNS | WNL | WNL | NT | WNL, on recut | WNL, QNS |
| **CC corpus callosum:** | WNL | WNL | WNL | WNL | WNL, QNS | WNL | WNL | NT | NT | WNL, QNS |
| **Hippocampus:** | WNL | NT | WNL | WNL, QNS | WNL, QNS | WNL | WNL | NT | WNL QNS | WNL |
| **Cerebellum:** | QNS | NT | WNL | WNL | WNL, QNS | WNL | WNL, QNS | NT | NT | WNL |
| **Medulla** | QNS | WNL | WNL | WNL | WNL, QNS | WNL | WNL, QNS | NT | NT | WNL |
| **Pituitary:** | WNL | NT | QNS distalis | QNS distalis | WNL | NT | WNL | NT | NT | WNL, QNS |
| **Ears:** | WNL | WNL, QNS | WNL | WNL | WNL, QNS | WNL | WNL | WNL | WNL, QNS | WNL, QNS |
| **Eyes:** | WNL, pigmented | WNL, pigmented | WNL, pigmented, QNS | WNL, pigmented | WNL, QNS | WNL, QNS | WNL, pigmented | WNL, QNS: pigmented | WNL, pigmented | WNL, pigmented |
| **Harderian:** | WNL | WNL | WNL | WNL | WNL | WNL | WNL | WNL | WNL | WNL |
| **Oral/molars:** | WNL | WNL | WNL | WNL | WNL | WNL | WNL | WNL, QNS | WNL, QNS | WNL, QNS |
| **Incisors:** | WNL | WNL | WNL | WNL | WNL | WNL | WNL | WNL | WNL | WNL, WNS |
| **Nose:** | WNL | WNL | WNL | WNL | WNL | WNL | WNL | WNL | WNL | WNL |
| **Other:** |  |  |  |  |  | Thymus <1mm ectopic, WNL |  |  |  |  |

**Abbreviations:** Aniso - Liver hepatocyte anisocytosis anisokaryosis (common finding increases with age); CL - Ovary Corpus luteum; CW - consistent/compatible with; DG – Degeneration; EMH - extramedullary hematopoiesis; FCC - follicular center cells; GC - Germinal center; Infilt - infiltration (mononuclear unless otherwise qualified); Inflam - inflammation (mononuclear unless otherwise qualified); LN – Lymph node; MF – Multifocal; M:E - Marrow Myeloid:erythroid ratio (subjective); NSF - No significant findings (unremarkable); NT/NP – No tissue / Not present; QNS – Quantity not sufficient; R:W – Spleen Red: White pulp ratio (subjective); SC – subcutaneous; SCH – Adrenal subcapsular hyperplasia; Vacuol – Cytoplasmic vacuolation; WNL/NSF – Within normal limites / No significant findings (unremarkable); XZ – adrenal X zone (vacuolations expected in females at certain ages); ZG – adrenal zona glomerulosa; ZF – adrenal zona Fasciculata; ZR – adrenal zona Redicularis

**Scoring:** 0 = normal / unremarkable / NSF / WNL; 1 = mild; 2 = moderate; 3 = Marked / severe; H = High; L = Low
